# EYE movement abnormalities in Parkinson’s disease: optometric screening and rehabilitation

**DOI:** 10.1016/j.prdoa.2026.100468

**Published:** 2026-06-18

**Authors:** Reshu Yadav, Dayashankar Rastogi, Shivam Kumar, Partha Chowdhury, Eric Austin

**Affiliations:** a Department of Optometry, School of Health Sciences, CSJM University, Kanpur, Uttar Pradesh, India; b Department of Optometry, School of Allied Health Sciences, Galgotias University, Greater Noida, India 293201; c Robert and Sons Eyecare Ltd., Greater Accra Region, Ghana

**Keywords:** Parkinson's disease, Eye movement abnormalities, Saccades, Vergence, Smooth pursuit, Biomarkers, Visual rehabilitation

## Abstract

**Background:**

Parkinson's disease (PD) is increasingly recognized as a multisystem disorder in which eye movement abnormalities provide important diagnostic and prognostic insights.

**Objective:**

This review synthesizes current evidence on oculomotor disturbances in PD, their clinical relevance, and their potential as biomarkers for early detection and disease monitoring.

**Methods:**

A structured narrative review was conducted using PubMed, Scopus, and Web of Science databases (up to July 2025). Although a systematic search strategy was applied, findings were synthesized narratively due to heterogeneity in study designs and outcomes.

**Results:**

Across studies, PD patients demonstrated prolonged saccadic latency, reduced vergence amplitudes, and impaired smooth pursuit, often correlating with disease severity. Several abnormalities were detectable in early-stage disease, preceding overt motor dysfunction. Interventions such as prisms, vergence exercises, and vision therapy showed potential benefits in alleviating diplopia and improving visual function. Quantitative eye-tracking measures emerged as promising objective biomarkers for disease monitoring.

**Discussion:**

Oculomotor assessment offers significant clinical implications, from supporting early diagnosis and differential recognition of parkinsonian syndromes to guiding rehabilitative strategies that enhance visual quality of life. However, heterogeneity of methodologies and limited longitudinal data constrain generalizability.

**Conclusions:**

Eye movement abnormalities represent a valuable, underutilized tool in PD care. Standardization of assessment protocols, integration with neuroimaging, and validation through large-scale longitudinal studies are essential to establish oculomotor metrics as reliable biomarkers for clinical practice.

## Highlights

• Parkinson's disease exhibits characteristic abnormalities in saccades, smooth pursuit, and vergence eye movements.
• Oculomotor deficits differ from normal aging and may support early detection of Parkinson's disease.
• Retinal OCT and eye-movement analysis provide complementary structural and functional biomarkers.
• Quantitative oculomotor parameters may aid differentiation of PD from atypical parkinsonian syndromes.
• AI-based eye-tracking offers promising potential for objective screening and digital biomarker development.

## 1 Introduction

Parkinson's disease (PD) is a progressive, idiopathic neurodegenerative disorder primarily affecting the nigrostriatal dopaminergic pathway, with hallmark pathology including α-synuclein accumulation and Lewy body formation. [1], [2] Although historically characterized by cardinal motor symptoms tremor, rigidity, bradykinesia, and postural instability there is growing consensus that non-motor features constitute a substantial component of the disease burden. [3] Among these, visual and oculomotor disturbances are gaining increased attention due to their high prevalence, early onset, and measurable impact on functional autonomy. [4] Visual dysfunction in PD extends beyond reduced visual acuity and includes complex deficits in ocular motor control, which are often underrecognized in routine clinical assessments. [5] These include abnormalities in saccadic execution, smooth pursuit tracking, vergence mechanisms, fixation stability, and blink dynamics. [6] Such disturbances often manifest before or independently of overt motor signs, indicating their potential utility as prodromal biomarkers. Moreover, these oculomotor impairments compromise patients' ability to perform essential visual tasks, such as reading, scanning the environment, and maintaining visual fixation, directly impacting quality of life and increasing the risk of falls and accidents. Saccadic eye movements rapid, conjugate shifts in gaze that direct the fovea to targets of interest are frequently impaired in PD. [7] These saccadic anomalies typically present as hypometric responses, prolonged latency periods, and diminished peak velocities. [8] These patterns are believed to arise from basal ganglia dysfunction and disrupted connectivity between the superior colliculus, frontal eye fields (FEF), and supplementary eye fields (SEF). In PD, voluntary saccades during cognitive tasks such as reading or visual search may become effortful, imprecise, or delayed. Importantly, saccadic performance is often more compromised during anti-saccade or memory-guided tasks, reflecting deficits in executive control. [9] Smooth pursuit eye movements, which enable continuous tracking of moving objects, are likewise significantly affected in PD. Reduced pursuit gain defined as the ratio of eye velocity to target velocity is a consistent finding, often accompanied by compensatory catch-up saccades that interrupt smooth gaze. [10] Pursuit deficits are attributed to impaired cortico-basal ganglia circuitry, involving the dorsolateral prefrontal cortex, parietal lobe, and cerebellum. [11] Unlike reflexive eye movements, smooth pursuit relies on integrative cortical functions, which are disrupted in early PD, sometimes even in the absence of motor symptoms. [12] These changes suggest early cortical involvement and highlight smooth pursuit analysis as a valuable tool in early-stage diagnostics. Convergence insufficiency is another prominent oculomotor anomaly in PD, reported in up to 30 to 40% of cases. Patients may report symptoms such as intermittent diplopia, asthenopia, blurred near vision, or difficulty concentrating during reading. [13] The pathophysiology is likely multifactorial, involving dopaminergic depletion in midbrain structures such as the supraoculomotor area, which controls vergence, as well as impaired coordination between the medial rectus subnuclei. [14] Notably, convergence insufficiency in PD may partially respond to dopaminergic medications but often remains sub optimally corrected, necessitating additional rehabilitative measures. [15] Fixation instability is also well-documented, manifesting as increased occurrence of square-wave jerks, micro saccades, and fixational drift. These involuntary movements are particularly prominent during attempted steady gaze and are associated with reduced frontal lobe inhibition and cerebellar modulation. Such instability can interfere with reading, targeting, and sustained attention, and often correlates with neurocognitive decline, especially executive dysfunction. [16] Eye-tracking technologies have revealed increased variability in fixation duration and precision among PD cohorts, particularly in later stages or in individuals with coexisting cognitive impairment. [17] An additional layer of complexity arises from non-motor ocular manifestations, including reduced blink rate, blepharospasm, impaired pupillary response, and dry eye symptoms. [18] These factors contribute to visual discomfort, photophobia, and compromised visual endurance. The reduced blink frequency in PD, resulting from impaired basal ganglia output, contributes to tear film instability, exacerbating symptoms of dry eye disease and further impairing visual clarity. [18] Despite the significant burden of visual and oculomotor disturbances in PD, their recognition remains limited in standard neurological practice. The underdiagnosis may stem from the prioritization of motor symptom management and the lack of systematic visual assessments during routine care. [19] However, structured optometric evaluations offer an accessible, non-invasive avenue to detect and monitor these visual abnormalities. Clinical tools such as dynamic retinoscopy, prism cover testing, Maddox rod evaluation, vergence amplitude assessment, and computerized oculography allow for precise quantification of these deficits. [20] Importantly, many of these visual disturbances are amenable to rehabilitative intervention. For example, convergence insufficiency can be addressed using base-in prism lenses, vision therapy exercises to enhance fusional reserves, and reading aids. Saccadic training programs, including software-based protocols or guided visual scanning exercises, have shown efficacy in improving ocular control and compensatory strategies. [21] Visual cueing methods and contrast enhancement techniques may help mitigate some visual deficits by leveraging preserved pathways. Furthermore, collaborative care models involving neurologists, optometrists, and rehabilitation specialists can ensure that visual complaints are neither minimized nor misattributed to cognitive or psychological factors. [22] From a pathophysiological perspective, these visual anomalies underscore the broader systems-level disruption in PD that extends well beyond the nigrostriatal axis. [23] Involvement of cortical, cerebellar, and brainstem structures, as well as cholinergic and serotonergic systems, is evident in the genesis of oculomotor symptoms. Hence, visual abnormalities in PD serve as both a functional impairment and a reflection of widespread neurodegeneration. Their temporal evolution may also mirror the disease's trajectory, offering potential markers for disease staging and prognosis [24]. In conclusion, eye movement abnormalities in PD are pervasive, clinically significant, and highly informative. Their presence offers valuable insights into the underlying neural networks affected in the disease and provides a unique opportunity for early detection, objective monitoring, and personalized intervention. As PD is increasingly recognized as a multisystem disorder, incorporating optometric screening and rehabilitative protocols into comprehensive care pathways is not only justified but essential for optimizing patient outcomes. [25]

Despite substantial research on oculomotor abnormalities in Parkinson's disease, current literature often treats these deficits in isolation, lacking integration of their progression, functional relevance, and therapeutic implications within a unified clinical context. Furthermore, the role of optometric assessments in early diagnosis and phenotypic staging across PD subtypes remains underexplored. This review consolidates current evidence on ocular motor dysfunctions in PD [26], emphasizing their diagnostic and rehabilitative significance. It further proposes an interdisciplinary, protocol-driven approach to incorporate eye movement analysis into routine PD care. By positioning oculomotor deficits as both clinical features and potential biomarkers of multisystem neurodegeneration, this work underscores the need for standardized visual screening in PD management a perspective not yet reflected in prevailing neurological guidelines.

## 2 Screening and rehabilitation approaches

Early identification and management of oculomotor dysfunction in Parkinson's disease (PD) are essential to reduce visual disability and improve functional independence. A structured optometric screening protocol should include- Dynamic retinoscopy, prism cover test, and Maddox rod for detecting binocular misalignment and convergence insufficiency. Vergence range measurement and computerized eye tracking for analysing saccades, pursuits, and fixation control. Blink rate observation and tear film tests to evaluate dry eye symptoms associated with reduced blink frequency. Although high-quality randomized controlled trials specific to Parkinson's disease remain limited, current recommendations are supported by emerging clinical studies and established neuro-optometric rehabilitation literature. [7].

Many of these visual disturbances are amenable to rehabilitation: Prism lenses and vision therapy (e.g., Brock string, pencil push-ups) help address convergence issues. [15] Saccadic and pursuit training software improves gaze control and reading efficiency Fixation stability exercises and visual attention tasks enhance executive eye movement control. [7], [27], [28] Blink training and lubrication therapy support ocular surface health and comfort. A collaborative care model involving neurologists, optometrists, and rehabilitation specialists ensures comprehensive management. Incorporating these assessments into routine PD care offers a practical, non-invasive way to improve both diagnostic accuracy and quality of life an area still underrepresented in current guidelines. Although PD-specific randomized rehabilitation trials remain limited, current recommendations are supported by emerging clinical studies and extrapolated neuro-optometric rehabilitation evidence.

Additional structured searches specifically captured studies related to retinal OCT biomarkers, AI-based oculomotor diagnostics, and differential oculomotor profiles in PSP/MSA, which are now incorporated into the formal evidence synthesis.

## 3 Methodology

This study was conducted as a structured narrative review. A systematic and reproducible search strategy was applied to identify relevant literature; however, the review was not designed or conducted as a formal systematic review, as it was not prospectively registered and does not include meta-analysis. PRISMA 2020 principles were used to guide reporting transparency.

Primary objective To synthesize evidence on oculomotor (saccades, smooth pursuit, vergence, fixation and blink) abnormalities in idiopathic Parkinson's disease (PD), their functional impact, and the effectiveness/feasibility of optometric screening and rehabilitation strategies. Secondary objectives To describe variability by disease stage and cognitive status, assess which oculomotor metrics are most promising as biomarkers, and identify gaps and recommendations for clinical screening protocols.

Timeframe and language the primary search focused on literature published between January 2020 and July 2025 to capture recent advances in digital eye-tracking technologies, AI-based analytics, and contemporary biomarker research. Seminal earlier studies were additionally consulted for historical and mechanistic context, English language full-text articles.

**Search Strategy:** A structured search was conducted in PubMed, Scopus, and Web of Science using combinations of MeSH terms and keywords including: (“Parkinson's disease” OR “Parkinson's disease”) AND (“eye movement abnormalities” OR saccades OR vergence OR smooth pursuit OR fixation instability OR oculomotor dysfunction) AND (“optometric screening” OR rehabilitation OR eye tracking OR OCT OR artificial intelligence OR atypical parkinsonism OR PSP OR MSA). Example PubMed strategy: ((“Parkinson's disease”[MeSH]) AND (“Saccades”[MeSH] OR “Eye Movements”[MeSH] OR vergence OR smooth pursuit OR fixation)).

In addition to the primary oculomotor-focused search, extended structured searches were conducted to identify relevant literature on retinal OCT biomarkers, AI-based eye-tracking analytics, and differential diagnosis (PSP/MSA), which were incorporated as complementary evidence domains.

### 3.1 Eligibility criteria

#### 3.1.1 Inclusion

• Original clinical research (observational cohort, case–control, cross-sectional).
• Interventional trials (randomized or non-randomized) evaluating rehabilitative/optometric interventions. Diagnostic/accuracy studies assessing eye-movement metrics in PD.
• Systematic reviews, meta-analyses, and narrative reviews were included for contextual synthesis and reference cross-checking; however, they were not classified as primary evidence studies in the analysis.
• Experimental human studies with primary oculomotor outcomes.
• (Quantitative studies only; qualitative studies may be summarized narratively if directly relevant.)
• Adults (≥18 years) with clinically diagnosed idiopathic Parkinson's disease (any stage).
• Studies that report separate data for idiopathic PD if mixed parkinsonian cohorts are used.

#### 3.1.2 Exclusion

• Secondary parkinsonism or atypical parkinsonian syndromes unless idiopathic PD data are separably reported.
• Case reports with <3 subjects, conference abstracts without full text, editorials, commentaries, and animal-only studies.
• Studies lacking primary oculomotor data (e.g., those reporting only retinal imaging without ocular motor measures).
• Non-English full texts (unless translation is available).

### 3.2 Quality assessment

Methodological quality of included primary studies was assessed using the Joanna Briggs Institute (JBI) Critical Appraisal Checklist appropriate to each study design. Narrative reviews were included only for contextual synthesis and were not subjected to formal bias scoring.

### 3.3 Data extraction

The following variables were extracted from eligible studies: author/year, country, study design, sample size, PD stage, oculomotor domain assessed, assessment technology used, key findings, and study limitations.

**PRISMA Flow Diagram:**

**Figure:**
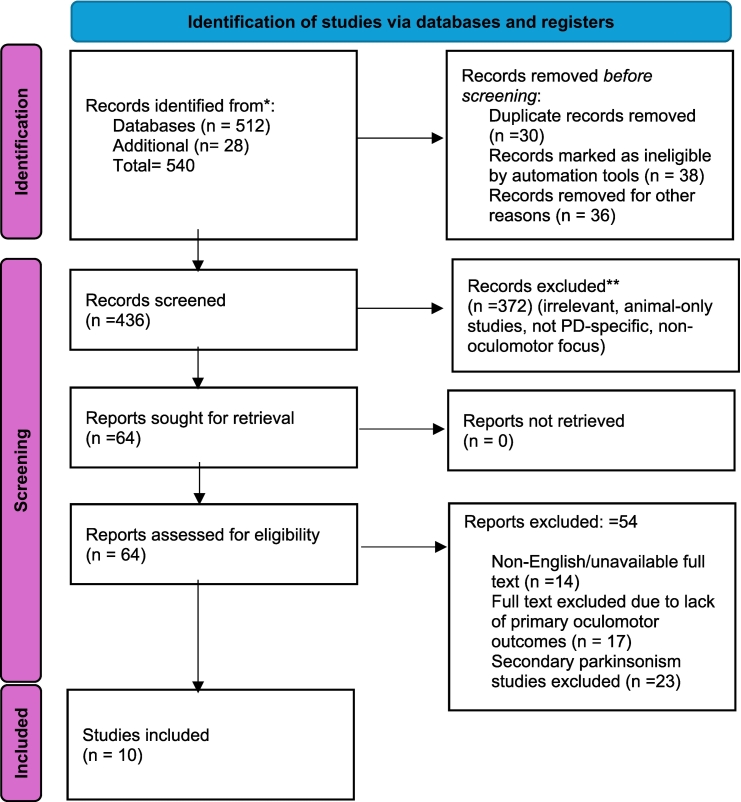

## 4 Results

### 4.1 Study selection and characteristics

The structured search identified a total of studies evaluating oculomotor abnormalities in Parkinson's disease (PD). Following screening and eligibility assessment, 10 primary studies met the inclusion criteria and were included in the formal evidence synthesis. These comprised observational studies, experimental studies, and interventional investigations focusing on quantitative oculomotor outcomes.

In addition to the primary oculomotor-focused search, extended structured searches identified relevant literature in complementary domains, including retinal imaging biomarkers, artificial intelligence (AI)-based eye movement analytics, and differential diagnosis of parkinsonian syndromes. Due to differences in study design, objectives, and outcome measures, these domains were analyzed separately and are presented as complementary evidence within the Results to enhance clinical and translational interpretation.

The characteristics of included primary studies are summarized in Table 1. (See Table 2, Table 3, Table 4.)

**Table 1:** Functional impact of eye movement abnormalities in Parkinson's disease.

Eye movement abnormality	Functional impact in PD
Saccadic dysfunction	Delayed reading & impaired target shifting
Smooth pursuit deficit	Poor tracking of moving stimuli
Convergence insufficiency	Blurred near vision, diplopia
Fixation instability	Difficulty in visual fixation & attention
Reduced blink rate	Visual discomfort & dry eye symptoms

**Table 2:** Summary of included primary studies.

No.	Authors & year	Review design	Methodology	Identified gaps	Key results & conclusion
1	Stefanescu et al., 2024 [29]	Retrospective	Eye-tracking of visually guided saccades in 62 PD patients correlated with cognition and demographics	No longitudinal follow-up limited scope of oculomotor analysis	Saccadic metrics linked with cognitive status and demographics, highlighting eye-tracking as a promising, non-invasive PD monitoring tool.
2	Shaikh AG, Sun YR, Ghasia FF (2023) [30]	Narrative Review	Clinical and research studies on eye movement changes in Parkinson's patients were analyzed.	Parkinson's patients show abnormal saccades, fixation instability, and vergence issues. Eye movement issues relate to brain areas like basal ganglia and brainstem.	Lack of real-world tracking data, no standardized clinical protocols, and limited use of portable eye-tracking tools
3	Fooken J., Patel P., Jones CB, McKeown MJ, Spering M. (2022). [31]	Experimental Study	Eye-tracking tasks comparing Parkinson's patients and controls using visual targets (moving vs stationary)	Parkinson's patients showed poor eye movement to static targets but normal tracking of moving ones. Suggests alternate motor pathways are preserved.	Small sample, lab-based tasks, lacks real-world application and long-term follow-up.
4	Sekar AT-N, Kaski D. (2025) [32]	Narrative Review	Reviewed studies on eye movement abnormalities in neurodegenerative diseases	Eye movement changes (like saccadic and pursuit deficits) can help differentiate conditions like PD and Alzheimer's.	No standard clinical protocols, limited normative data, and lack of real-world studies.
5	Li H., Zhang X., Yang Y., Xie A. (2023) [8]	Narrative review	Summarized clinical and research studies on eye movements in Parkinson's disease	PD patients show abnormal saccades, fixation issues, and impaired pursuit. Eye tracking may help in diagnosis and monitoring.	No standard protocols, limited real-world validation, and small sample sizes in most studies.
6	Sonawane et al., 2023 [33]	Narrative Review	Synthesized data on ocular and retinal changes in PD	Need for reliable biomarkers limited long-term data.	PD is associated with diverse ocular and visual deficits that may act as early indicators and guide targeted interventions.
7	Antoniades & Spering, 2023 [34]	Narrative Review	Cross-species synthesis of neurophysiological and behavioural evidence related to oculomotor control in Parkinson's disease	Lack of standardized, disease-specific eye-movement markers; limited clinical integration of oculomotor tools	Eye movement abnormalities in PD reflect underlying neural deficits and treatment status. While not diagnostic in isolation, they hold value as part of a multimodal biomarker approach for disease staging and monitoring.
8.	Bronstein et al., 2022 [35]	Book Chapter Review	Descriptive analysis of oculomotor and vestibular impairments in PD based on clinical and experimental evidence	Lack of real-world data, limited ecological validity of lab-based findings	Eye movement abnormalities are well-characterized in lab settings, but their relevance to daily function is underexplored. Field-based studies are essential for clinical translation.
9.	Nieto-Escamez et al., 2023 [26]	Narrative Review	Synthesized literature on ocular and visual-perceptual impairments in PD	Incomplete mechanistic understanding; limited longitudinal data	Visual dysfunction in PD spans motor and perceptual domains. Though linked to structural and neurochemical changes, real-world impact remains under-investigated
10.	Gupta et al. 2023 [36]	Observational Study	Video-oculography in PD patients vs. controls to assess alignment & disparity-driven vergence	Small sample, no longitudinal data	PD patients showed frequent binocular misalignment and impaired vergence (slower initiation, lower gain), independent of motor stage, highlighting early binocular screening need.

**Table 3:** Oculomotor abnormalities in Parkinson's disease vs normal aging.

Parameter	Normal aging	Parkinson's disease (PD)	Clinical significance
Saccadic latency	Mildly increased	Markedly prolonged	Early indicator of basal ganglia dysfunction
Saccadic accuracy (gain)	Slight hypometria	Significant hypometria with variability	Impaired gaze shifting; affects reading
Anti-saccade performance	Mild errors	Increased error rate, poor inhibition	Reflects executive dysfunction
Smooth pursuit gain	Slight reduction	Markedly reduced with catch-up saccades	Difficulty tracking moving objects
Fixation stability	Generally preserved	Increased instability, square wave jerks	Reduced visual attention
Vergence (NPC)	Mild decline	Convergence insufficiency common	Diplopia, near vision problems
Blink rate	Slight reduction	Significantly reduced	Dry eye, ocular discomfort

**Table 4:** Oculomotor features in PD vs atypical Parkinsonian syndromes.

Feature	Parkinson's disease (PD)	Progressive supranuclear palsy (PSP)	Multiple system atrophy (MSA)
Vertical saccades	Relatively preserved (early)	Severely impaired (early hallmark)	Mild to moderate impairment
Saccadic velocity	Mild–moderate reduction	Markedly slowed	Variable
Saccadic accuracy	Hypometric	Hypometric with restriction	Variable
Smooth pursuit	Impaired	Severely impaired	Impaired (cerebellar pattern)
Gaze palsy	Absent (early stages)	Present (supranuclear)	Rare
Nystagmus	Rare	Rare	Common (gaze-evoked)
Clinical utility	Supportive biomarker	Strong diagnostic marker	Helps differentiate subtype

### 4.2 Primary oculomotor findings in Parkinson's disease

#### 4.2.1 Saccadic dysfunction

Saccadic abnormalities were the most consistently reported finding across included studies. PD patients demonstrated prolonged saccadic latency, hypometric saccades, and increased variability in amplitude and velocity. Impairments were more pronounced in cognitively demanding paradigms such as anti-saccade and memory-guided tasks, suggesting involvement of executive control networks. Several studies also reported correlations between saccadic parameters and disease severity as well as cognitive status.

#### 4.2.2 Smooth pursuit abnormalities

Smooth pursuit deficits were frequently observed, characterized by reduced pursuit gain and increased reliance on compensatory catch-up saccades. Task-dependent variability was noted, with some studies reporting partial preservation of pursuit during specific stimulus conditions, indicating possible recruitment of alternative neural pathways in early disease stages.

#### 4.2.3 Vergence dysfunction

Vergence abnormalities, particularly convergence insufficiency, were reported in multiple studies. These deficits were associated with symptoms such as diplopia, blurred near vision, and difficulty with reading tasks. Quantitative assessments revealed reduced vergence gain, delayed initiation, and impaired binocular coordination, often occurring independently of motor disease stage.

#### 4.2.4 Fixation instability and blink abnormalities

Fixation instability was characterized by square-wave jerks, micro-saccadic intrusions, and increased fixation variability, reflecting impaired gaze stabilization mechanisms. Additionally, reduced blink rate was consistently reported, contributing to ocular surface dysfunction, dry eye symptoms, and visual discomfort.

#### 4.2.5 Effectiveness of optometric and rehabilitative interventions

Limited but emerging evidence from interventional and observational studies suggests that optometric interventions, including prism correction, vergence exercises, and oculomotor training programs, may improve visual function and reduce symptom burden in PD patients. However, the overall evidence base remains limited by small sample sizes and lack of large randomized controlled trials.

### 4.3 Evidence from extended search domains

#### 4.3.1 Retinal imaging biomarkers (OCT)

Extended searches identified multiple studies utilizing optical coherence tomography (OCT), which consistently demonstrated thinning of the retinal nerve fiber layer (RNFL) and ganglion cell inner plexiform layer (GC-IPL) in PD patients. These structural changes were frequently correlated with disease severity, duration, and cognitive impairment, supporting their potential role as non-invasive biomarkers of neurodegeneration.

#### 4.3.2 Artificial intelligence-based oculomotor analytics

Studies employing AI and machine learning approaches demonstrated promising ability to classify PD based on quantitative eye movement parameters, including saccadic latency, variability, and smooth pursuit performance. These models highlight the potential of eye-tracking data as objective digital biomarkers, particularly for early detection and disease monitoring.

#### 4.3.3 Differential diagnosis of Parkinsonian syndromes

Comparative studies examining oculomotor profiles across parkinsonian disorders revealed distinct patterns. Progressive supranuclear palsy (PSP) was characterized by early impairment of vertical saccades and gaze palsy, whereas multiple system atrophy (MSA) showed greater involvement of cerebellar pathways with impaired smooth pursuit and gaze-evoked nystagmus. In contrast, PD patients typically exhibited relatively preserved vertical gaze in early stages but demonstrated deficits in voluntary and task-dependent eye movements.

Overall, the synthesized evidence demonstrates that PD is associated with multidimensional oculomotor dysfunction, involving saccadic, pursuit, vergence, and fixation systems. These abnormalities are clinically relevant, functionally significant, and measurable using objective eye-tracking technologies. Complementary evidence further supports the integration of structural imaging, computational analytics, and differential diagnostic profiling to enhance the clinical utility of oculomotor assessment in PD. Narrative reviews included in Table 1 are presented for contextual synthesis and are not considered primary evidence studies.

The characteristics and key findings of included primary studies are summarized in Table 1.

## 5 Discussion

The present structured narrative review synthesizes evidence from primary oculomotor studies alongside complementary domains identified through extended structured searches.

Eye movement control is a sensitive indicator of neural integrity, and accumulating evidence demonstrates that Parkinson's disease (PD) is associated with a wide spectrum of oculomotor abnormalities. Across the studies reviewed, consistent deficits were observed in saccadic performance, smooth pursuit, fixation stability, and vergence control, supporting the notion that eye movements represent a reliable window into basal ganglia and brainstem dysfunction.

Recent systematic evidence by Gibbs et al. (2024) further supports the ecological relevance of naturalistic eye movement paradigms in PD, emphasizing their value in detecting subtle functional impairments beyond laboratory-based tasks. [37]

Saccadic abnormalities were the most consistently reported finding. [29], [30] documented prolonged saccadic latency, increased variability, and reduced accuracy, with several parameters correlating with cognitive decline and disease severity. These findings suggest that saccades may reflect both motor and non-motor aspects of PD. However, methodological heterogeneity including variations in recording techniques and patient selection limits direct comparison across studies.

Smooth pursuit impairments were also evident, although their manifestation was task-dependent. [31] It demonstrated that PD patients showed poor tracking of static stimuli but relatively preserved pursuit of moving targets, suggesting that compensatory neural circuits may remain functional in the early stages. This highlights the importance of differentiating between pursuit subtypes when interpreting clinical findings.

Fixation instability and micro-saccadic intrusions were described in both observational and review-based studies, with potential implications for reading, visual exploration, and daily functioning. While these abnormalities are not specific to PD, their presence in conjunction with other oculomotor deficits may enhance diagnostic accuracy.

Vergence dysfunction particularly convergence insufficiency emerged as another robust feature of PD. [36] It identified early binocular misalignment and reduced vergence gain, while [33] emphasized its clinical relevance, given that patients frequently complain of diplopia, reading difficulty, and ocular fatigue. Unlike saccadic or pursuit deficits, vergence abnormalities often appear independent of motor stage, suggesting that they may serve as early diagnostic markers.

The collective evidence underscores the potential of eye-tracking technologies as objective, non-invasive biomarkers. Automated video-oculography, as employed by [36], enables quantitative characterization of eye movement patterns, providing a feasible tool for both clinical and research settings. Moreover, comparative reviews [32], [34] have suggested that oculomotor profiling may help distinguish PD from other neurodegenerative disorders such as Alzheimer's disease or progressive supranuclear palsy. This diagnostic differentiation is of particular relevance in early disease stages, when motor features may overlap.

In addition to core oculomotor findings, extended evidence domains identified through structured searches included retinal imaging biomarkers, AI-based eye movement analysis, and differential diagnostic applications. Optical coherence tomography (OCT) studies consistently reported thinning of the retinal nerve fiber layer (RNFL) and ganglion cell–inner plexiform layer (GC-IPL), with correlations to disease severity and cognitive status. Emerging studies utilizing artificial intelligence and machine learning demonstrated the ability to classify Parkinson's disease based on quantitative oculomotor parameters such as saccadic latency, variability, and pursuit gain. Furthermore, comparative oculomotor profiling studies highlighted distinct patterns that may aid in differentiating Parkinson's disease from atypical parkinsonian syndromes such as progressive supranuclear palsy and multiple system atrophy. These domains were incorporated as complementary evidence to enhance the clinical and translational relevance of the review.

Nevertheless, significant limitations temper the current evidence base. Most included studies were cross-sectional with relatively small sample sizes, limiting generalizability. There is also considerable variability in methodology, including differences in stimulus paradigms, outcome measures, and analytic frameworks. Furthermore, few studies have integrated oculomotor findings with other biomarkers (e.g., imaging, genetics, electrophysiology), which could enhance mechanistic understanding and clinical applicability. Longitudinal data tracking the progression of eye movement abnormalities alongside motor and cognitive decline remain scarce.

From a clinical perspective, the recognition of oculomotor abnormalities carries both diagnostic and rehabilitative implications. Early identification of convergence insufficiency and vergence deficits may guide targeted interventions such as prism correction, convergence exercises, or vergence therapy, potentially improving quality of life. Similarly, characterizing saccadic and pursuit dysfunction could inform adaptive strategies for reading and navigation. Integration of oculomotor assessments into standard neurological evaluation may therefore add value beyond traditional motor scales.

In conclusion, the evidence reviewed highlights that oculomotor abnormalities are prevalent, multifaceted, and clinically meaningful in PD. While saccadic, pursuit, fixation, and vergence deficits have been reliably demonstrated, further work is needed to standardize protocols, validate biomarkers across diverse populations, and establish longitudinal trajectories. Future research should focus on combining oculomotor measures with neuroimaging, cognitive profiling, and therapeutic interventions to fully exploit their potential as diagnostic and monitoring tools in PD.

#### 5.1.1 Oculomotor changes in normal aging versus Parkinson's disease

Distinguishing oculomotor abnormalities attributable to Parkinson's disease (PD) from those associated with physiological aging is essential for improving diagnostic accuracy. Normal aging is accompanied by modest alterations in ocular motor control, including mild increases in saccadic latency, slight reductions in peak velocity, and a gradual decline in smooth pursuit gain. These changes are typically subtle, slowly progressive, and rarely result in significant functional impairment in healthy older adults. In contrast, PD is characterized by more pronounced and functionally relevant disturbances. Saccadic deficits extend beyond simple latency changes and include hypometria, increased variability, and impaired performance in higher-order tasks such as anti-saccades, reflecting executive dysfunction. Smooth pursuit abnormalities in PD are more marked, with reduced gain and frequent corrective catch-up saccades. Vergence dysfunction, particularly convergence insufficiency, is also more prevalent and clinically significant, often contributing to symptoms such as diplopia and reading difficulty. [39], [40]

Overall, while aging and PD share certain overlapping features, the severity, pattern, and functional consequences of oculomotor abnormalities differ substantially. These distinctions emphasize the importance of using age-matched normative data and standardized testing paradigms to enhance the specificity of oculomotor assessments in clinical and research settings.

#### 5.1.2 Retinal and structural ocular biomarkers in Parkinson's disease

In addition to functional abnormalities, structural retinal changes have emerged as important biomarkers in Parkinson's disease. Optical coherence tomography (OCT) studies have consistently demonstrated thinning of the retinal nerve fiber layer (RNFL) and ganglion cell–inner plexiform layer (GC-IPL) in individuals with PD. These findings are thought to reflect underlying neurodegenerative processes, potentially linked to dopaminergic dysfunction and α-synuclein-related pathology.

Several studies have reported associations between retinal thinning and clinical parameters such as disease severity, duration, and cognitive impairment, suggesting that OCT may provide a non-invasive method for monitoring disease progression. However, variability in imaging protocols and inconsistencies in reported outcomes currently limit its routine clinical application. [8]

Importantly, retinal imaging complements oculomotor assessment by providing structural information, whereas eye movement analysis reflects functional neural integrity. The integration of these modalities offers a multimodal biomarker approach that may improve early detection, enhance disease stratification, and support longitudinal monitoring in PD. [8]

### 5.2 Oculomotor signatures in Parkinson's disease versus atypical parkinsonian syndromes

Oculomotor abnormalities, although common in Parkinson's disease, are not disease-specific and should be interpreted in the context of differential diagnosis. Quantitative analysis of eye movement parameters can provide valuable insights for distinguishing PD from atypical parkinsonian syndromes, including progressive supranuclear palsy (PSP) and multiple system atrophy (MSA) [40].

PSP is typically characterized by early impairment of vertical saccades, particularly reduced velocity and range, often progressing to supranuclear gaze palsy. In contrast, patients with PD generally exhibit relatively preserved vertical gaze in early disease stages, with deficits more prominent in voluntary and cognitively demanding saccadic tasks. MSA, on the other hand, may show less pronounced saccadic abnormalities but greater involvement of cerebellar pathways, leading to impaired smooth pursuit and gaze-evoked nystagmus. [39]

These differences highlight the importance of detailed parametric evaluation, including measures such as saccadic latency, velocity, gain, and error rates in anti-saccade tasks. Incorporating such quantitative metrics into clinical assessment can enhance diagnostic precision and support differentiation between parkinsonian syndromes.

#### 5.2.1 Artificial intelligence and digital biomarkers in oculomotor assessment

Advances in artificial intelligence (AI) and machine learning have significantly expanded the potential of oculomotor analysis in Parkinson's disease. Eye-tracking systems generate high-dimensional datasets that can be analyzed using computational approaches to identify disease-specific patterns. Machine learning models have demonstrated promising accuracy in classifying PD patients based on features such as saccadic latency, variability, and smooth pursuit performance. Deep learning techniques further enable the detection of complex, non-linear relationships within eye movement data, offering potential for early identification of subtle abnormalities that may not be detectable using conventional analysis. These approaches are particularly relevant for identifying prodromal or early-stage disease. [27], [28]

In addition, the development of portable and wearable eye-tracking devices allows for real-world data collection, enabling continuous monitoring outside laboratory environments. Integration with digital health platforms and telemedicine systems may facilitate scalable screening and longitudinal disease tracking.

Despite these advances, challenges remain, including the need for standardized data acquisition protocols, large annotated datasets, and external validation across diverse populations. Nonetheless, AI-driven approaches represent a promising direction for developing objective and scalable digital biomarkers in PD.

#### 5.2.2 Impact of comorbidities on oculomotor function in Parkinson's disease

Oculomotor dysfunction in Parkinson's disease is influenced not only by primary neurodegenerative processes but also by coexisting comorbidities, which may affect both assessment and interpretation. Cognitive impairment, particularly involving executive function, has been associated with deficits in voluntary saccadic control, including increased error rates in anti-saccade paradigms and reduced fixation stability. [27]

Ocular surface disorders, such as dry eye disease, are also common in PD due to reduced blink rate and autonomic dysfunction. These conditions contribute to visual discomfort, blurred vision, and reduced visual endurance, potentially exacerbating perceived oculomotor symptoms. In addition, psychiatric comorbidities such as depression and anxiety may influence attentional processes and visual performance. [8]

Pharmacological treatment further contributes to variability, as dopaminergic therapy may improve certain oculomotor parameters while having limited effects on others. This variability reflects the involvement of both dopaminergic and non-dopaminergic pathways in oculomotor control. A comprehensive understanding of these comorbid factors is essential for accurate interpretation of findings and for developing individualized management strategies. Future studies should aim to account for these variables and investigate their interaction with disease-specific mechanisms.

### 5.3 Clinical implications

Eye movement abnormalities in Parkinson's disease hold significant clinical value. Early signs such as vergence insufficiency and saccadic latency may support earlier diagnosis and aid in differentiating PD from other neurodegenerative disorders. Targeted interventions like prisms, vergence exercises, and visual training can help manage diplopia, fixation instability, and reading difficulties. Quantitative eye-tracking offers objective biomarkers for monitoring disease progression and treatment response. Incorporating oculomotor assessment into multidisciplinary care, alongside neurologists and ophthalmologists, can improve both functional outcomes and quality of life for patients with PD.

### 5.4 Limitations and future directions

Current evidence on eye movement abnormalities in Parkinson's disease is limited by small sample sizes, heterogeneous methodologies, and lack of standardized assessment tools. Many studies are cross-sectional, restricting insights into long-term progression. Future research should focus on large, longitudinal studies using uniform eye-tracking protocols, integration with neuroimaging, and exploration of digital, portable eye-tracking tools for routine clinical use. Establishing robust oculomotor biomarkers could improve early detection, track disease progression, and guide individualized rehabilitation strategies. The inclusion of complementary domains such as AI and retinal biomarkers, while systematically searched, was primarily synthesized narratively and should be interpreted as supportive rather than primary outcome evidence.

## 6 Conclusion

Eye movement abnormalities in Parkinson's disease are not merely ancillary findings but represent sensitive, functionally relevant biomarkers of the disease's multisystem involvement. Evidence from clinical, neurophysiological, and neuroimaging studies underscores their potential role in early detection, phenotypic differentiation, and monitoring of disease progression. The heterogeneity in presentation ranging from saccadic hypometria and pursuit deficits to convergence insufficiency and fixation instability reflects both dopaminergic and non-dopaminergic pathway dysfunction, explaining the variable responsiveness to current pharmacological and surgical interventions. Importantly, these visual motor disturbances frequently compound sensory deficits, amplifying the burden on daily activities such as reading, navigation, and object tracking. Despite the depth of laboratory characterization, translation into routine care remains limited by the absence of standardized protocols, normative reference values, and widely accessible assessment tools. The integration of structured optometric screening, supported by portable eye-tracking technologies and telemedicine-ready platforms, offers a feasible and cost-effective pathway to bridge this gap. Coupled with targeted rehabilitation strategies including vision therapy, prism correction, and environmental adaptation such integration can enable earlier intervention, guide personalized management, and improve functional outcomes. Future multicentre longitudinal studies are essential to establish the prognostic value of these measures and to validate their role in comprehensive, multidisciplinary PD care.

## CRediT authorship contribution statement

**Reshu Yadav:** Writing – original draft, Methodology, Investigation. **Dayashankar Rastogi:** Writing – review & editing, Visualization, Methodology. **Shivam Kumar:** Resources, Formal analysis. **Partha Chowdhury:** Validation, Supervision. **Eric Austin:** Resources, Project administration, Investigation.

## Ethical statement

This study is a structured narrative review based on previously published literature and does not involve the collection of any primary data from human or animal subjects. Therefore, ethical approval and informed consent were not required. All included studies in this review were assumed to have obtained the appropriate ethical clearances as reported by their respective authors.

## Declaration of competing interest

The authors declare that there are no conflicts of interest relevant to the content of this manuscript. No financial or non-financial relationships exist that could be perceived to influence the work reported in this paper. The authors have received no funding, grants, or other support for the preparation of this manuscript.

All authors have reviewed and approved the final version of the manuscript and agree with its submission to the Parkinsonism and Related Disorders.
